# Morphological and lipid metabolism alterations in macrophages exposed to model environmental nanoplastics traced by high-resolution synchrotron techniques

**DOI:** 10.3389/fimmu.2023.1247747

**Published:** 2023-09-08

**Authors:** Federica Zingaro, Alessandra Gianoncelli, Giacomo Ceccone, Giovanni Birarda, Domenico Cassano, Rita La Spina, Chiara Agostinis, Valentina Bonanni, Giuseppe Ricci, Lorella Pascolo

**Affiliations:** ^1^ Physics Department, University of Trieste, Trieste, Italy; ^2^ Elettra-Sincrotrone Trieste, Trieste, Italy; ^3^ European Commission, Joint Research Centre (JRC), Ispra, Italy; ^4^ Institute for Maternal and Child Health, IRCCS Burlo Garofolo, Trieste, Italy; ^5^ Department of Medical, Surgical and Health Science, University of Trieste, Trieste, Italy

**Keywords:** polypropylene, polyvinyl chloride, nanoplastics (NPs), macrophages (M1), lipid metabolism, XRF, FTIR, CdSe-QDs

## Abstract

The release of nanoplastics (NPs) in the environment is a significant health concern for long-term exposed humans. Although their usage has certainly revolutionized several application fields, at nanometer size, NPs can easily interact at the cellular level, resulting in potential harmful effects. Micro/Nanoplastics (M/NPs) have a demonstrated impact on mammalian endocrine components, such as the thyroid, adrenal gland, testes, and ovaries, while more investigations on prenatal and postnatal exposure are urgently required. The number of literature studies on the NPs’ presence in biological samples is increasing. However, only a few offer a close study on the model environmental NP–immune system interaction exploited by advanced microscopy techniques. The present study highlights substantial morphological and lipid metabolism alterations in human M1 macrophages exposed to labeled polypropylene and polyvinyl chloride nanoparticles (PP and PVC NPs) (20 μg/ml). The results are interpreted by advanced microscopy techniques combined with standard laboratory tests and fluorescence microscopy. We report the accurate detection of polymeric nanoparticles doped with cadmium selenide quantum dots (CdSe-QDs NPs) by following the Se (L line) X-ray fluorescence emission peak at higher sub-cellular resolution, compared to the supportive light fluorescence microscopy. In addition, scanning transmission X-ray microscopy (STXM) imaging successfully revealed morphological changes in NP-exposed macrophages, providing input for Fourier transform infrared (FTIR) spectroscopy analyses, which underlined the chemical modifications in macromolecular components, specifically in lipid response. The present evidence was confirmed by quantifying the lipid droplet (LD) contents in PP and PVC NPs-exposed macrophages (0–100 μg/ml) by Oil Red O staining. Hence, even at experimental NPs' concentrations and incubation time, they do not significantly affect cell viability; they cause an evident lipid metabolism impairment, a hallmark of phagocytosis and oxidative stress.

## 1 Introduction

Plastics refer to a heterogeneous group of synthetic materials with carbon–carbon backbones produced by the polymerization of different monomers, with variable addition of functional additives to satisfy the desired product features (1). The inevitable degradation of plastics produces, therefore, a complex mixture of micro- and nanoplastics (M/NPs), universally detected with a broad range of shapes, sizes, chemical composition, and concentration in the air (2), food (3), freshwater (4), and sea (5). It was calculated that 8.3 billion metric tons of plastics were produced worldwide by 2020, and 51 trillion MPs are floating in the oceans. According to the European Chemicals Agency (ECHA), NPs are polymeric particles smaller than 1 μm, whereas MPs are between 1 μm and 5 mm. Among a broad classification criterion (size, shape, and chemical composition), M/NPs can be classified according to their source: primary M/NPs are directly manufactured for commercial purposes and can be found as micro- and nano-beads in personal care products, plastic fibers, or nurdles; whereas secondary M/NPs come from the fragmentation of larger plastics (6).

It is widely accepted that environmental exposure to M/NPs may have a long-lasting impact on subjects’ health (7–9). Although solid epidemiological data on the actual occurrence and accumulation in the human body are still partial because of the analytical difficulties in their detection, many toxicological effects are proposed by the increasing number of scientific studies (10–12), mainly in biological models. Recently, microplastic particles have been detected for the first time in the placentas of unborn children; therefore, there is growing concern about the effects of this pollution on embryonic, fetal, and general reproductive health. Although the mechanisms of toxicity are still unknown, it is conceivable that plastics and associated chemicals could cause long-term damage and perturb the normal development of the immune system in the fetus (13).

Because of the complexity of polymeric and organic composition, size, and shape, and the possibility that these particles may be associated with other pollutants and microbes on their surfaces, the assessment of human risks is an intricate and challenging mission that cannot be fulfilled with only standard approaches, given the great need for procedures that combine characterization methods (microscopy and spectroscopy) to the classical toxicological investigations.

Although the biological risk resulting from exposure to NPs more than to MPs is widely accepted, as far as we know, some studies are reporting consolidated analytical methods capable of characterizing and investigating them in human organisms.

Because of their size, nanosized particles are more likely to enter cells and interact with intracellular organelles, proteins, and macromolecules (14, 15). They are crucial for understanding NP cell accumulation and related toxicological outcomes to short- and long-term exposure.

From a nanotoxicology experience, we know that, in human cells, nanoparticles (NPcs) interact with the phospholipidic bilayer according to their physicochemical characteristics (size, shape, composition, polarity, surface charge, etc.) (16). When cell–NPcs interaction occurs, NPcs can enter the cell membrane via specific (clathrin- and caveolin-mediated phagocytosis) or non-specific membrane pathways (macro-pinocytosis and pinocytosis) depending on NPs dimensions and cell type. The professional phagocytotic cells (macrophages, dendritic cells, and neutrophils) play an essential role as a physiological barrier to remove NPcs by the phagocytosis pathway, which is an active-dependent process mediating the engulfment of non-self-particles larger than 500 nm in size (17). Macrophagic cell lines are widely exploited to investigate the nanomaterials’ biocompatibility and internalization mechanisms (18) and the inflammatory responses (19), as well to study nanomaterials in bioimaging for human diseases, such as cancer, diabetes, myocardial infarction, and atherosclerosis (20).

As for other pollutants, various immune cells (innate immune cells like macrophages and neutrophils) and epithelial cells of barrier organs collaborate in the defense action of the body to the non-self-agents upon exposure (21). Macrophages represent a heterogeneous population of myeloid cells of the innate immune system and are involved in several physiological and pathological processes, such as inflammation and infection. It is well known that, owing to their convenient availability, monocytic cell lines at varying degrees of differentiation are often used as surrogates of macrophages (22); therefore, the monocytic THP-1 cell line is conventionally used as an *in vitro* system for producing macrophages as for this study.

Macrophages can be differentiated into two phenotypes M1, which mediates the pro-inflammatory response, and M2, characterized by regulatory functions in tissue repair or remodeling. In this regard, M1 phenotype macrophages were chosen for the present research study because of their primary immune response against exogenous agents and subsequent cellular interactions (23).

Importantly, THP-1 monocyte-polarized macrophages (M1) showed the highest phagocyting ability after exposure to nano- and micro-particulates, and they have already been used in some studies to start investigating M/NPs toxicity mechanisms (9). It has been recently demonstrated that the THP-1 cell line is the best model, compared to other leukocytic cell lines, to phagocytise polystyrene (PS) MPs after 24–48 h of exposure time (8).

Using light fluorescence microscopy, Stock et al. already demonstrated that different-sized PS MPs (1, 4, and 10 μm) were uptaken by THP-1 cells physiologically differentiated into M0, M1, and M2 after 24–72 h (24). Interestingly, Jeon et al. employed scanning electron microscopy (SEM) to follow the interaction between THP-1 cells and 100-μm-sized polystyrene and polypropylene (PP) MPs. M/NPs trigger cytotoxicity, with pro-inflammatory response being higher for nano-sized plastics than for MPs (7).

One of the main difficulties in toxicological studies with plastics is the need for efficient and sensitive techniques to visualize and characterize the polymeric material in a complex matrix. Indeed, conventional optical and fluorescence microscopes can only support sample preparation and higher-resolution measurements. However, the use of advanced techniques, such as vibrational and chemical spectroscopies, is highly demanding, like synchrotron-radiation-based ones reported in this work. Apart from the optical detection limit, energy dispersive X-ray spectrometry (EDS) can only provide the chemical information at 1 μm lateral resolution, and SEM/transmission electron microscopy (TEM) techniques allow the detection of NPs but not their chemical composition, which is instead possible with Raman spectroscopy (13). Recently, scanning transmission X-ray microscopy (STXM) combined with near-edge X-ray absorption fine-structure spectroscopy has been exploited to visualize and characterize 100-nm-sized NPs in environmental and food matrices, thanks to the high-resolution images and spectral information (25).

For the model studies, many laboratories proposed utilizing M/NPs labeled with fluorescent probes with successful results following their biodistribution. However, they may suffer from photobleaching, imprecise detection in human fluids, and frequent aggregate formation in biological media (26). An increasing number of scientific studies using commercially available PS nanospheres of different sizes (27–29) have been published to unravel the toxicity of NPs (27–29). However, studies must be considered cautiously since the cited polymer is not the most abundant in the environment. The use of PP and PVC NPs, instead, is primarily representative of plastic fragments commonly found, for instance, on the sea surface (5), in sampled lakes, ponds, and surface flooding (30), or in food packages (31).

As an alternative solution to characterize the NPs’ biological activities, a different model of labeled NPs is proposed in this work. We support the use of chemically synthesized and fully characterized PP and PVC NPs labeled with CdSe-QDs performed by Cassano et al. (32). The as-synthetic route allows us to obtain NPs at tuneable size in the nano range (50–350 nm), with included inorganic species, CdSe-QDs, that confer specific traceability under light fluorescence microscopy and spectroscopy.

Since the QDs are included in the polymer’s core, the labeling is highly stable, as demonstrated by the performed analytical techniques (Energy-dispersive X-ray spectroscopy and inductively coupled plasma – mass spectrometry) by Cassano et al. (32). Moreover, the CdSe-QDs PP and PVC NPs were demonstrated to be stably dispersed in Dulbecco’s modified Eagle’s medium (DMEM), in phosphate-buffered saline solution 1× (DPBS) at alkaline and neutral pH conditions.

Owing to the peculiar chemical composition, the labeling with QDs, emitting in the red range (645 ± 75 nm), in light fluorescence microscopy allows combining the NPs detection by the elemental imaging *in vitro* models under advanced synchrotron-based micro-spectroscopies.

To date, by exploiting a specific range of soft X-ray photons (200–2,000 eV energy range) as a source, STXM can provide topographic information of cells in response to NPs exposure at sub-micron spatial resolution. Simultaneously, the morphological changes can be correlated with the spatial distributions of light elements, such as Na, O, C, and Mg, which support understanding the cell’s healthy condition. Thus, we present the use of STXM coupled with low-energy X-ray fluorescence (LEXRF) for the visualization and understanding of NPs fate at the cellular level (33): by tuning the excitation energy, it is possible to follow the fluorescence X-ray emission of the Se L-line and localize the CdSe-QDs-labeled NPs, while STXM absorption and phase contrast images fully characterize the cell stress-induced morphological changes in PP and PVC NPs-exposed macrophages. In addition, our results were complemented with Fourier transform infrared (FTIR) spectroscopy, a non-destructive vibrational technique able to characterize the whole cell and the cellular region in close contact with NPs at the macromolecule level (lipids, proteins, and nucleic acids) looking for any macromolecular modifications in the NPs-exposed cells, significant to unravel cellular toxicological impacts upon nano-environmental particle exposure, with particular attention to lipid metabolism (34).

## 2 Materials and methods

### 2.1 Nanoplastics synthesis and characterization

Polymeric labeled NPs were synthesized at the Joint Research Centre (JRC), Ispra, Italy, as illustrated in a previous paper (32).

Briefly, polypropylene and polyvinyl chloride nanoplastics (PP and PVC NPs) were prepared by the oil-in-water emulsion technique by dissolving 30 mg of plastic polymers (Sigma-Aldrich) in 3 g of toluene in a 50-ml flask, mixed at 100°C for 1 h to dissolve the pellets. QdotTM 655 ITKTM (ThermoFisher) organic quantum dots (200 μl) were inserted in the toluene solution. Afterwards, a boiling ultra-pure MilliQ water solution (27 ml) containing 7.5 mg of the dissolved surfactant sodium cholate (Sigma-Aldrich) was added to the hydrophobic phase and the two separating phases were homogenized using an Ultra-Turrax T25 (IKA) at 16,000 rpm for 2 min, ultrasonicated (Vibra-Cell Ultrasonic Liquid Processors, vCX 130) at 40% amplitude for 2.5 min, and cooled in an ice-water bath for 3 min. Five-micrometer polyether-sulfone membrane syringe filters filtered the reaction product to obtain the desired NPs size, while the organic phase was successfully evaporated using the rotavapor. The synthetic yield was calculated by weighing the freeze-dried powder. QDs NPs were dispersed in MilliQ and stored at 4°C under dark conditions (32).

### 2.2 Cell culture and sample preparation

Human monocytic THP-1 cells (ATCC TIB-202) were maintained in a sub-confluent state in complete culture medium, RPMI 1640 (Life Technologies, MI, Italy) supplemented with 10% (v/v) fetal bovine serum (Life Technologies, MI, Italy) and 1% (v/v) penicillin/streptomycin (Life Technologies, MI, Italy) under standard cell culture conditions (37°C, 5% CO_2_, and 95% humidity). The cells were cultured in a 75-cm^2^ Falcon flask for 2–3 days and then seeded onto silicon carbide (SiC) with 200-nm-thick membranes (Silson Ltd, Warwickshire, UK) in 24 multi-well plates. For THP-1 polarization, cells were seeded at 1 × 10^5^ cells/ml in the complete culture medium (CM) with phorbol-12-myristate 13-acetate (PMA) (Thermo Fisher Scientific, MA, USA) to a final concentration of 15 ng/ml. They were incubated at 37°C, 5% CO_2_ for 48 h, to be activated into resting macrophages (RM), and washed with phosphate-buffered saline, Dulbecco’s formula (PBS) supplemented with Ca^++^ and Mg^++^ (Life Technologies), and the CM was replaced with a PMA-free CM to control the macrophages’ differentiation. RM was polarized into M1 macrophages by the addition of the following stimuli: IFN-γ (500 U/ml) and LPS (100 ng/ml) (PeproTech, London, UK) for 24 h. The different conditioned cells were incubated with CM containing the two different NPs labeled with CdSe-QDs at a final concentration of 20 μg/ml for 72 h. Control M1 cells were grown under the same conditions but were not exposed to NPs. The samples were then fixed at room temperature with a 4% aqueous paraformaldehyde solution for 20 min in the dark and washed with PBS and Milli‐Q water before the analyses.

### 2.3 Cell viability test (MTT assay)

Cells were seeded in a 96-well Falcon plate and treated with different concentrations of labeled and non-labeled PP and PVC NPs (0, 1, 5, 20, and 50 μg/ml), and Staurosporine 2 μM (Sigma Aldrich, Milan, Italy) as a positive control. After 24 h of incubation, they were activated and polarized into the M1 phenotype and then treated with the testing concentrations of NPs for 72 h. MTT reagent (3-(4,5-dimethylthiazol-2-yl)-2,5-diphenyl tetrazolium bromide) (Sigma-Aldrich, MI, Italy) (10 μl) was added to each conditioned well in triplicate, incubated for 4 h at 37°C, 5% CO_2_. The formazan crystals were visible through the light microscope; thus, they were dissolved in dimethyl sulfoxide (DMSO) (Euroclone, MI, Italy), and the corresponding absorbance was read using an ELISA Microplate Reader (Bio-Rad, Hercules, CA, USA) at 570 nm wavelength. The data were collected in triplicate for each condition, the negative control of the reagent used (MTT) was subtracted and all the values were normalized for the control cells (100%) using GraphPad software.

### 2.4 Oil Red O staining

THP-1 cells (10^4^ cells/ml) were seeded in a 96-well plate and treated with different concentrations of CdSe-QDs PP and PVC NPs (0, 5, 10, 20, 50, and 100 μg/ml). After 24 h of incubation, monocytes were activated and polarized into the M1 phenotype and then treated with the testing concentrations of NPs for 24 and 72 h. At this point, cells were washed twice with PBS, and 100 μl of PFA, 4% in PBS, was added to each well and left for 20 min in the dark. Then, sterile water was gently rinsed into the wells. Isopropanol (100 μl) was added to each well and left to sit for 2–5 min. Oil Red O (Acros Organics, Geel, Belgium) working solution (100 μl) was added to the wells, and the multi-well was slowly rotated to favor the spread of the solution along the cells. After 5 min, the wells were washed with sterile water until the water ran clear. Isopropanol (200 μl) was added to each well, whose 150 μl was transferred to a new well plate for quantification. The corresponding absorbance was read using an ELISA Microplate Reader (Bio-Rad, Hercules, CA, USA) at 450 nm wavelength. The *t*-test calculated the significance of the data using GraphPad software.

### 2.5 Fluorescence microscopy

For each experimental condition, two to three cells among the samples grown on the same or different SiC windows were selected by performing light fluorescence microscopy, to be afterwards analyzed by X-ray microscopy at Elettra Sincrotrone Trieste. SiC windows were mounted on the sample holder for fluorescence microscopy (BioTek Cytation 5 Cell Imaging Multimode Reader, Agilent, CA, USA), operating both in bright field mode and with Texas Red ^®^ filter cube (excitation wavelength 560 ± 55 nm and emission wavelength 645 ± 75 nm). Images were acquired at 10× and 20× magnification objectives for each experimental condition and were carried out using FiJi software (35).

### 2.6 Scanning transmission X-ray microscopy and low-energy X-ray fluorescence

STXM and LEXRF were performed at the TwinMic beamline (33, 36) at Elettra Sincrotrone Trieste (Trieste, Italy). The TwinMic microscope was operated in the scanning transmission mode (STXM), and the beam was focused on the sample through a zone plate (600 μm diameter and 50 nm outermost zone width), delivering a micrometric or sub-micrometric probe size. Samples were raster-scanned perpendicularly to the incoming monochromatic beam. At the same time, a fast readout charge-coupled device (CCD) camera collected the transmitted X-rays (37), and an eight-silicon drift detector-based XRF system acquired the emitted fluorescence photons (36, 38). The obtained absorption and phase contrast images outlined the morphological sample features at sub-micrometer length scales, while the simultaneous detection of LEXRF correlated the elemental distribution to the morphology. Elemental distribution has been obtained with PyMCA software (39) by deconvolving and fitting the XRF spectra. For high-resolution images operating in the STXM mode, a photon energy of 1.2 keV was used, with a spot size of approximately 200 nm and a dwell time of 60 ms. Parameters were then changed to acquire XRF maps of a smaller area, using a photon energy of 1.7 keV to excite and obtain optimal emission conditions for the element of primary interest, Se, with a spot size of 490 nm and a dwell time of 10 s per pixel for XRF mapping, and a CCD dwell time of 60 ms for STXM imaging. Each map lasted approximately 8−12 h, depending on the dimensions of the scanned area. We mapped approximately two to three cells per SiC window.

### 2.7 Fourier transform infrared micro-spectroscopy and imaging

FTIR micro-spectroscopy measurements were performed at the Chemical and Life Science branch of the SISSI-Bio beamline, Elettra Sincrotrone Trieste (Trieste, Italy) (40), using a Hyperion 3000 Vis-IR microscope (15× condenser/objective) and an MCT detector coupled with a Vertex 70v interferometer (Bruker Optics GmbH, Ettlingen, Germany). FTIR measurements were gathered in transmission mode in the MIR region (4,000–800 cm^−1^ at 4 cm^−1^ spectral resolution). For each condition, 40 to 300 single spectra (averaging 512 scans per spectrum and 1 min per spectrum) were acquired in both cytoplasmatic and nuclear regions. Background spectra were collected for each measure in a cell-free area with the same parameters. In addition, cellular imaging was performed using a 64 × 64 pixel focal plane array (FPA) detector, collecting two to three maps per condition averaging 256 scans (20 min per image) with 4 cm^−1^ spectral resolution and a pixel size of 2.6 microns (15× condenser/objective). Data analyses were conducted employing OPUS software (Bruker Optics, Billerica MA, US) for water vapor correction and then Quasar (http:\\quasar.codes) for spectra processing: baseline correction, cut, normalization when required, calculation of the band integrals and ratios, and principal component analysis (PCA). To screen the pixels belonging to empty areas that could affect the results, an intensity filter on the peak at 1,650 cm^−1^ was applied. Then, statistical analysis was carried out with OriginPro 2023 (Originlabs). For univariate analysis, the following band integrals were calculated: 2,800–3,000 cm^−1^ for lipids, peak height at 2,925 cm^−1^ for the CH_2_ asymmetric stretching, and at 2,960 cm^−1^ for the CH_3_ asymmetric stretching, 1,710–1,765 cm^−1^ for carbonyl group (CO), 1,716 cm^−1^ for free fatty acids (FFAs), 1,740 cm^−1^ for triacylglycerols (TAGs), and 1,700–1,480 cm^−1^ for proteins. Multivariate analysis was accomplished using Quasar. The second-order derivative of absorbance spectra was calculated by the Savitzky–Golay algorithm using 21 points of smoothing, a third-degree fitting polynomial function. Then, the data were normalized using vector normalization, and PCA was performed using 10 components. The results are shown as a scatterplot of PC1 vs. PC2 vs. PC3 and line plots of the respective loadings.

### 2.8 Statistical analysis

Mean and standard deviations were calculated for continuous variables, whereas percentages were reported for categorical variables. Sample data were analyzed by Student’s *t*-test. Analysis of different groups of data was performed using two-way analysis of variance (ANOVA). Results were expressed as mean ± standard deviations. *p*-values <0.05 were considered statistically significant. All statistical analyses were performed using GraphPad Prism software 10 (GraphPad Software Inc., La Jolla, CA, USA).

## 3 Results

### 3.1 Cell assays

#### 3.1.1 Effects of NPs on cell viability

**Figure 1:**
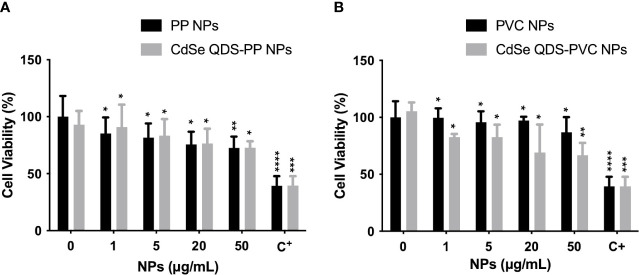
Analysis of cytotoxicity induced by NPs. Cellular viability percentage normalized over the controls (monocyte-macrophages M1 polarized) as a function of increased concentration (0–50 μg/ml) of not labeled and labeled PP NPs **(A)** and PVC NPs **(B)**, respectively. The positive control (C+) refers to the Staurosporine, apoptotic factor 2 μM (1:200). *p < 0.05, **p < 0.01, ***p < 0.001, ****p < 0.0001, black * (*t*-test method) versus untreated (0 μg/ml).

In order to evaluate the likely cytotoxicity induced by NPs, MTT assay was performed incubating the cells with increasing concentrations of NPs. The exposure was performed to assure that the used experimental condition of NPs (20 μg/ml) did not highly compromise the cellular functionality. The results obtained from the cell viability studies on M1 phenotype macrophages after 72 h of incubation with labeled (CdSe-QDs PP and PVC NPs) or non-labeled (PP and PVC NPs) NPs (0–50 μg/ml) are shown in **Figure 1**. The cell viability was kept at approximately 70% till the highest used NPs concentration (50 μg/ml) with no significant difference in the two polymers, in line with what was reported in the literature (8). As expected, the presence of QDs did not increase the toxicity of NPs, as shown in **Figures 1A, B**. The apoptotic factor, Staurosporine, was used as a positive control, demonstrating to decrease the cell survival down to 40%. The concentration corresponding to 20 μg/ml of CdSe-QDs PP and PVC NPs was selected for the uptake analyses based on the cell viability tests.

#### 3.1.2 Lipid droplet contents increase as a function of NPs concentration

**Figure 2:**
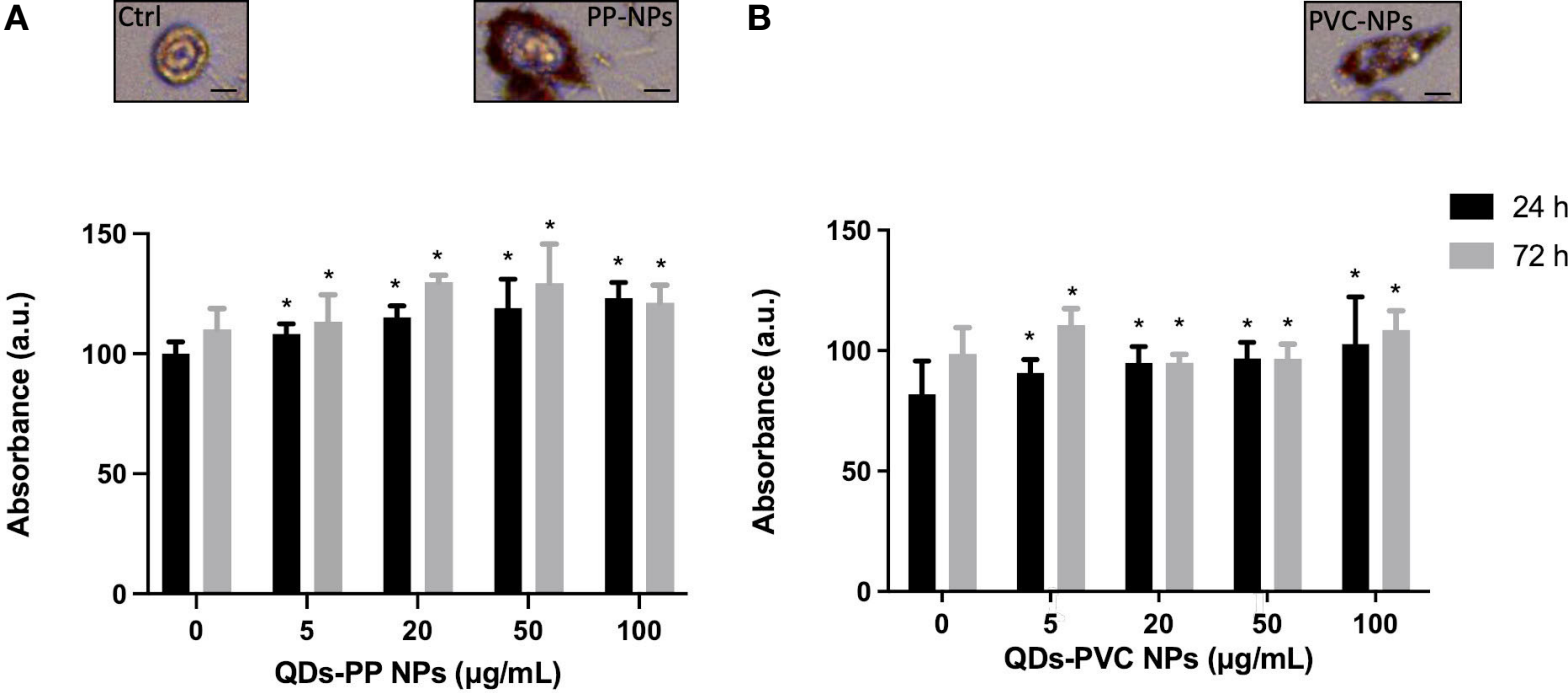
Analysis of lipid content at 24 or 72h by Oil Red O staining. Absorbance measured at 450 nm of the Oil Red O normalized over the controls (monocyte-macrophages M1 polarized) as a function of increased concentration (0–100 μg/ml) of CdSe-QDs PP NPs **(A)** and CdSe-QDs PVC NPs **(B)**, respectively. *p < 0.05, black * (*t*-test method) versus untreated (0 μg/ml). Insets show the red fluorescence images in the red range of selected cells as an example for the three treatment conditions. The scale bar is 5 μm.

To investigate the lipid metabolic response of M1 macrophages exposed to increased concentration of CdSe-QDs PP and PVC NPs (41), the Oil Red O staining was performed to identify a peculiar increase of LD formation. At the selected concentrations, ranging from 0 to 100 μg/ml of CdSe-QDs PP and PVC NPs (**Figures 2A, B**, respectively), the staining mentioned above was used to quantify the presence of the vesicles concerning the control, unexposed cells. After 24 h of incubation, the results show that all concentrations of NPs enhanced the LD contents with a slight, but statistically significant, linear increase from 100% in control cells to 130% in cells exposed to 100 μg/ml of CdSe QDs PP and PVC NPs. The LD quantification was also performed after 72 h of CdSe QDs PP and PVC NPs cell incubation, which revealed a linear consistency in the LD increase up to 20 μg/ml NPs, with a *p*-value <0.05. In contrast, an inversion occurred starting from 20 μg/ml of NPs concentration. It can be hypothesized that a more complex metabolic response takes place, such as apoptotic pathway activation. Again, the data confirmed that the 20 μg/ml concentration was the best compromise to characterize those lipidic macromolecular changes (FTIR).

The insets in **Figure 2** show the red fluorescence images of selected M1 macrophages as an example for the three treatment conditions at the experimental concentration of NPs used (20 μg/ml). It can be seen that the LDs contain fluorescence in the red range, absent in the control, untreated cells. By contrast, the LDs appear to localize mainly at the cell membrane level for treated macrophages.

### 3.2 Labeled NPs tracking in macrophages through red fluorescence

**Figure 3:**
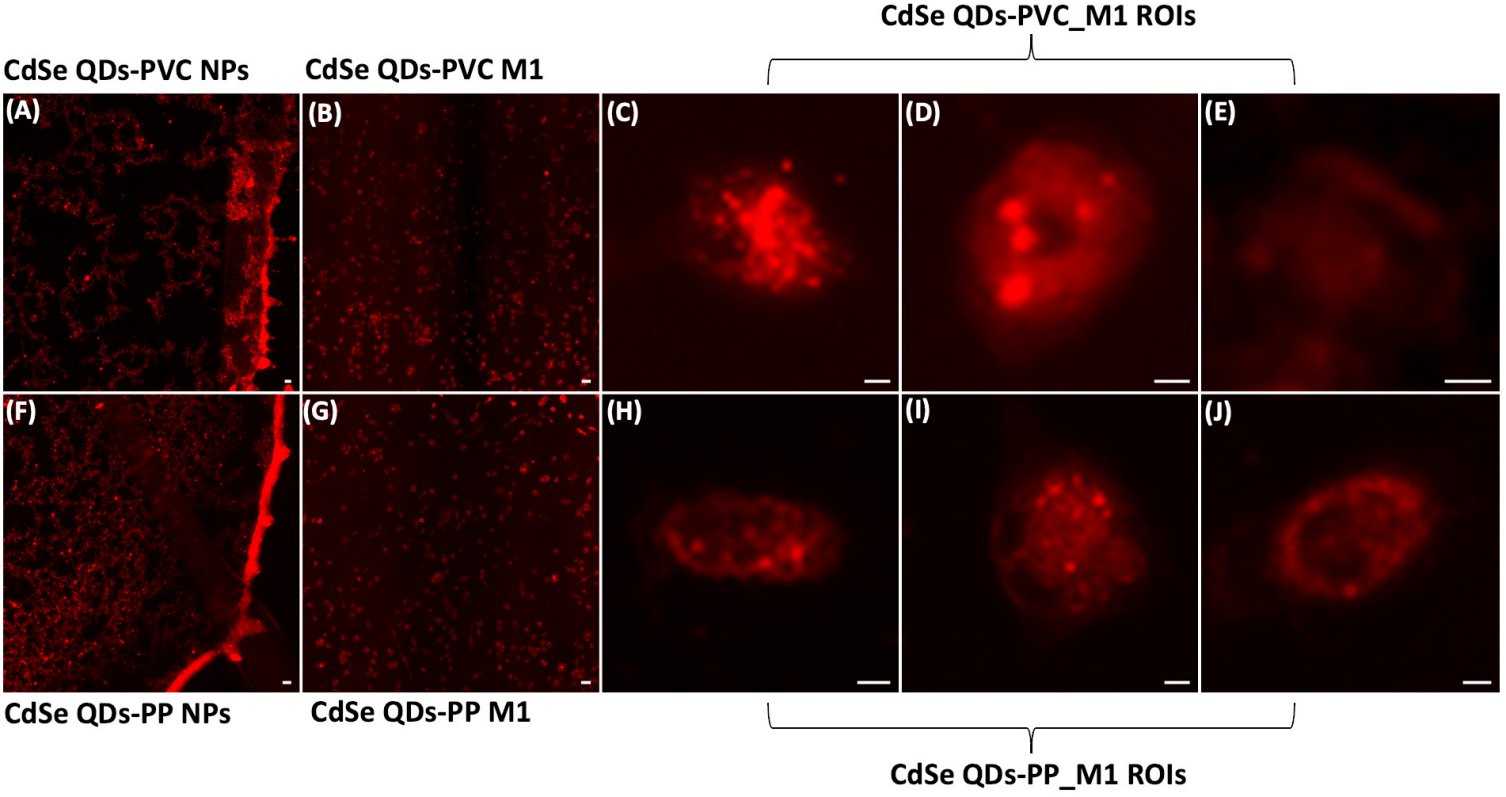
Fluorescence images characterize NPs localization. Fluorescence images of CdSe-QDs PVC NPs **(A)** and PP NPs **(F)** on silicon carbide windows with 200 nm thickness acquired with a Texas Red filter cube (excitation 560 ± 55 nm and emission 645 ± 75 nm) viewed using a fluorescence microscope at 10× magnification. Scale bar is 10 μm. Panels **(B, G)** present the M1 macrophagic cells exposed to 20 μg/ml of PVC NPs and PP NPs, respectively. The scale bar is 50 μm. Panels **(C–E)** and **(H–J)** show the regions of interest (ROIs) of the cells further analyzed. Brighter spots indicate the presence of PVC NPs and PP NPs, 20× magnification. The scale bar is 5 μm.

Light fluorescence microscopy analysis, able to detect the presence of NPs inside the cells, was preliminarily carried out to select the best cell samples for synchrotron-based studies. The light fluorescence images in **Figures 3A, F**, respectively, show the specific bright red emitting property of CdSe-QDs-PVC and -PP drops (1 mg/ml) deposited on 200-nm-thick SiC windows, acquired with the Texas Red filter cube (excitation 560 ± 55 nm and emission 645 ± 75 nm). Thus, this particular sized CdSe-QDs was confirmed to be an optimal labeling strategy to track NPs in cell samples (32). Despite a slight artifactual autofluorescence (red) of macrophages incubated with CdSe-QDs-PVC or -PP, visible in **Figures 3B, G**, respectively, the QDs-labeled NPs can be identified by the red spots at the highest brightness as depicted in **Figures 3C–E, H–J**. The light fluorescence images were extraordinarily supportive and used as a preliminary guide in the selection of two to three cells per sample to be analyzed at higher spatial resolution by the synchrotron-based technique, specifically LEXRF, to confirm the presence of NPs by following the emission L line of Se, one of the two components linked to NPs.

### 3.3 Cellular morphological changes in NPs-treated cells and detection of Se

**Figure 4:**
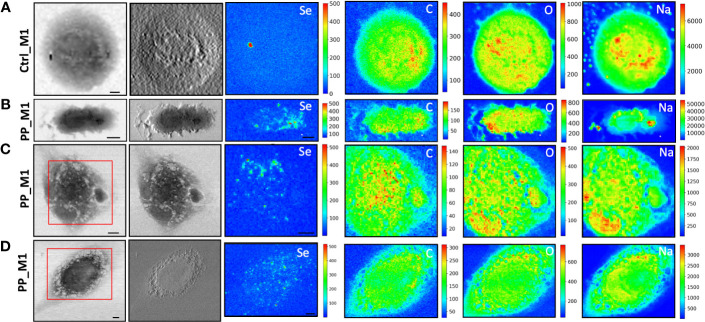
STXM coupled with LEXRF for PP NPs localization and cellular morphology. STXM absorption and phase contrast with μXRF maps of Se, C, O, and Na of monocyte-polarized M1 macrophages controls **(A)** and 20 μg/ml of CdSe-QDs PP NPs exposed **(B–D)**. The absorption and phase contrast images were measured at TwinMic beamline with 1.7 keV photon energy and 450 nm spatial resolution. The XRF maps show the elemental map distribution on selected areas (red square). The scale bars are 5 μm.

**Figure 5:**
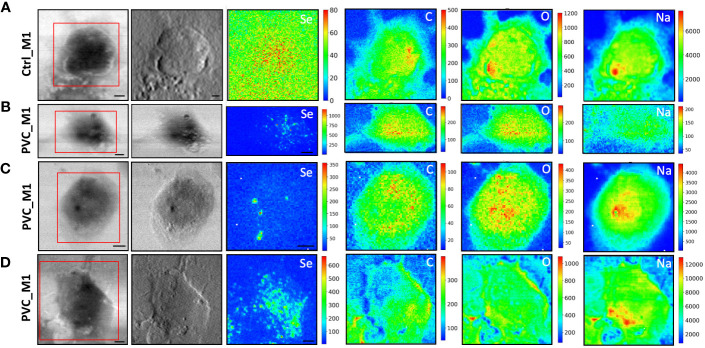
STXM coupled with LEXRF for PVC NPs localization and cellular morphology. STXM absorption and phase contrast with μXRF maps of Se, C, O, and Na of monocyte-polarized M1 macrophage controls **(A)** and 20 μg/ml of CdSe-QDs PVC NPs exposed **(B–D)**. The absorption and phase contrast images were measured at the TwinMic beamline with 1.7 keV photon energy and 450 nm spatial resolution. The XRF maps show the elemental map distribution on selected areas (red square). The scale bars are 5 μm.

To evaluate the NPs’ impacts on cellular morphology and to simultaneously track the NPs across the macrophages by following the Se L line of the label of NPs at sub-cellular spatial resolution, STXM and low-energy-X-ray fluorescence (LEXRF) were performed. The monocyte-polarized macrophage topographic information before and after the 72-h incubation of cells with NPs has been provided by STXM absorption and phase contrast images visible in **Figures 4**, **5**, respectively, for CdSe-QDs-PP and -PVC NPs. In **Figures 4A**, **5A**, the morphology of unexposed cells is shown, with a more absorbent nuclear region. Instead, when cells were exposed to NPs, especially PP, the formation of bright vesicles was often observed in the absorption and phase contrast images, like those reported in **Figures 4C, D**. Based on already published studies (41), the bright vesicles evidenced by STXM suggest LDs that frequently localize perinuclearly and at the plasma membrane. Contrary to fluorescence images (**Figure 3**), the presence of NPs aggregates at the cell surface cannot be directly inferred from absorption and phase contrast images.

More importantly, LEXRF analyses performed at 1.7 keV proved a suitable tool for tracing a component of NPs, selenium. This component is shown in the third column of **Figures 4**, **5**. The chemical distribution map of low-Z elements (C, O, Na, and Mg) was acquired and is displayed in the same figure. In this way, the cellular integrity and the specific localization of the NPs in the cells can be monitored.

The cells reported in **Figures 4B–D**, **5B–D** are the sameshown as the fluorescence image insets reported in **Figure 3**, whose red spot (NPs) localization is in good agreement with the selenium (QDs) distribution across the cell. Moreover, a possible internalization of NPs at submicron spatial resolution can be observed. Although the STXM coupled with LEXRF is not a 3D microscopy, the peculiar distribution of selenium in nanometric spots across the cells is suggestive of a cellular cytoplasmatic accumulation, with no evidence of interaction with the LDs.

The same measurement conditions were used as a negative control to acquire unexposed cell images which revealed no Se-related peak. These results do confirm the specificity of the Se signal for NPs presence. The hotspot appearing in **Figure 4A**, Se XRF map, corresponds to the Al contamination on the sample, as the Se L-line falls on the shoulder of the Al K-line.

### 3.4 FTIR imaging to reveal lipid metabolism impairment

To examine the cellular modifications at the macromolecular level induced by NPs exposure, FTIR microscopy and imaging was used.

**Figure 6:**
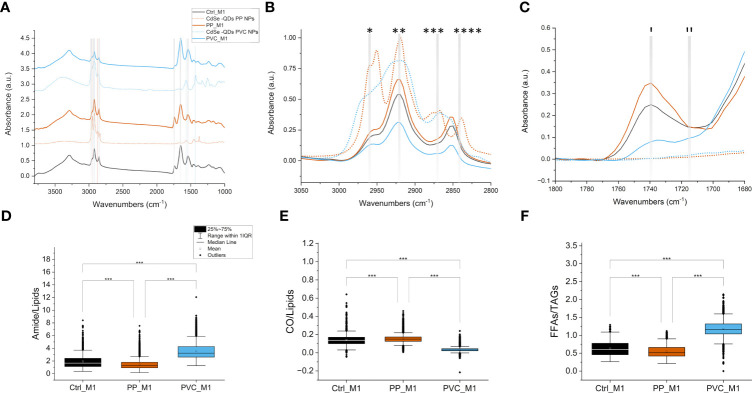
FTIR microscopy to investigate the macromolecular changes induced by NPs. Average spectra of 10 μl of QDs NPs (dashed lines) compared with average spectra of M1 macrophage cells (solid lines) treated with different QDs NPs, offset for clarity; the dashed vertical lines represent the characteristic signals of the PP-QDs NPs at 2,950 cm^−1^ and 2,870 cm^−1^, in orange, and at 2,920 cm^−1^, 1,568 cm^−1^, and 1,425 cm^−1^ for PVC-QDs NPs, in light blue, that do not overlap strongly with those of the cells. Spectra normalized from 0 to 1 over the whole range **(A)**. Detail of the 3,050–2,800 cm^−1^ range, cut from panel A without offset, characteristic of lipid aliphatic chain signals: *CH_3_ asymmetric stretching, **CH_2_ asymmetric stretching, ***CH_3_ symmetric stretching, and ****CH_2_ symmetric stretching. Same color code of **(A, B)**. Detail of the 1,800–1,680 cm^−1^ range, cut from panel A without offset, characteristic of lipid C=O ester signals: (I) ~1,737 cm^−1^ C=O of esters of TAGs and (II) ~1,716 cm^−1^ signal of the C=O of TAGs and/or aldehydes, same color code of the previous panels; solid lines denote spectra from cells, and dashed lines denote polymers **(C)**. Box plot of the FTIR interested bands of the amides over lipids **(D)**, CO over lipids **(E)**, and FFAs over TAGs **(F)**, ***p < 0.001, black *, and lines indicate significant differences calculated with the Tukey method. Data are presented as box 25%–75% range of the data; whiskers range within the 1st quartile, line: median line, square: mean, black diamonds: outliers.

In detail, FTIR microscopy characterized macromolecular changes in the cell samples exposed to NPs. Univariate analysis was accomplished to evaluate the spectral profiles of the different populations of cells and the values of specific band ratios. In **Figure 6A**, the normalized average spectra of the three groups of treated (PP_M1 and PVC_M1) and non-treated cells (Ctrl_M1) are shown (solid lines), compared with the spectra acquired on the labeled polymeric NPcs drops (dashed lines), prepared separately.

Specific signals from the NPs and the cells are highlighted in **Figure 6** as vertical dashed line and listed in **Table 1** for reference. Based on that, in our analyses, it was not possible to identify the NPs characteristic bands in the treated cells’ average spectra by looking at the solid orange line spectra for PP_M1, and the solid blue line spectra for PVC_M1 concerning the Ctrl_M1 in black, since the NPs size goes below the resolution limit for FTIR microscopy and their concentration within the samples is low. Thus, one must disentangle the FTIR vibrational changes of the polymeric material from those due to the cellular characteristic peaks of lipids (3,050–2,800 cm^−1^), proteins (1,700–1,480 cm^−1^), and nucleic acids (1,300–900 cm^−1^).

**Table 1:** Leading FTIR band assignments.

Spectral range [cm^−1^]	Assignment	Corresponding biomolecule	Reference
~2,950	*ν_as_* (CH_3_)	CH_3_ asymmetric stretching PP NPs	(42, 43)
~2,870	*ν_s_* (CH_3_)	CH_3_ symmetric stretching PP NPs	(42, 43)
~2,920	*ν_as_* (CH_2_)	CH_2_ asymmetric stretching PVC NPs	(44)
~1,568	*δ*(C-H)	CH_2_ bending PVC NPs	(44)
~1,425	*δ*(C-H)	CH_3_ symmetric bending PVC NPs	(44)
2,945–2,955	*ν_as_* (CH_3_)	CH_3_ asymmetric stretching mainly from lipids of THP-1_M_1_	(45)
2,920–2,930	*νa_s_* (CH_2_)	CH_2_ asymmetric stretching mainly from lipids of THP-1_M_1_	(45)
2,845–2,855	*ν_s_* (CH_2_)	CH_2_ symmetric stretching mainly from lipids of THP-1_M_1_	(46)
~1,737	*ν*(C=O)	CO stretching the esters mainly from TAGs	(47)
~1,716	*ν*(C=O)	CO stretching mainly from FFAs, ketones, and aldehydes.	(48)
1,670–1,614	Amide I: *ν*(C=O), *ν*(C-N)*, δ*(N-H)	Proteins: *β*-sheet structure at 1,632, random coil structure at 1,647	(49)
1,568–1,527	Amide II: *ν*(C-N), *δ*(N-H)	Proteins	(50)
1,481–1,431	*δ*(CH_2_, CH_3_)	Proteins, lipids	(51)
1,264–1,202	*ν_as_* (PO_2_^-^)	DNA/RNA backbone	(52)
1,105–1,069	*ν_s_* (PO_2_^-^)	DNA/RNA backbone	(53)

ν, stretching; δ, bending; s, symmetric; as, asymmetric.

In **Figure 6B**, a zoom in the 3,050–2,800 cm^−1^ range, characteristic of the stretching C-H vibrations of aliphatic chains, is reported with the spectra of the NPs (dashed lines) overlapping those of the cells (solid lines). The spectra of the cells are characterized by a different absorbance: PP_M1 cells indicate a higher intensity band in this spectral region, but no CH_3_ signals from the plastic can be directly seen (**Table 1**). PVC_M1 cells, instead, reveal signals with a lower intensity than the Ctrl_M1 and the PP_M1 cells.

**Figure 6C** presents the 1,800–1,700 cm^−1^ spectral range, where it is possible to identify the signal from C=O groups, either from the polar heads of lipids or from eventual oxidation by-products: (I) 1,737 cm^−1^ comes from C=O of esters of TAGs (47), while (II) the 1,716 cm^−1^ signal refers to the C=O of the FFAs and/or aldehydes, and ketones (54). From **Figure 6C**, it is possible to see that both NPs have no distinctive signals in this spectral region. In PP_M1 spectra, there is an increase of the ~1,737 cm^−1^ component of the C=O band concerning the control cells, whereas in PVC_M1, there is an increase of the ~1,716 cm^−1^ band. Any imbalance between the carbonyl signal at ~1,737 cm^−1^ and those from the aliphatic chains (C-H_x_ stretching) at 3,050–2,800 cm^−1^ could be caused by the presence of oxidative processes induced by the NPs exposure (55).

**Figure 7:**
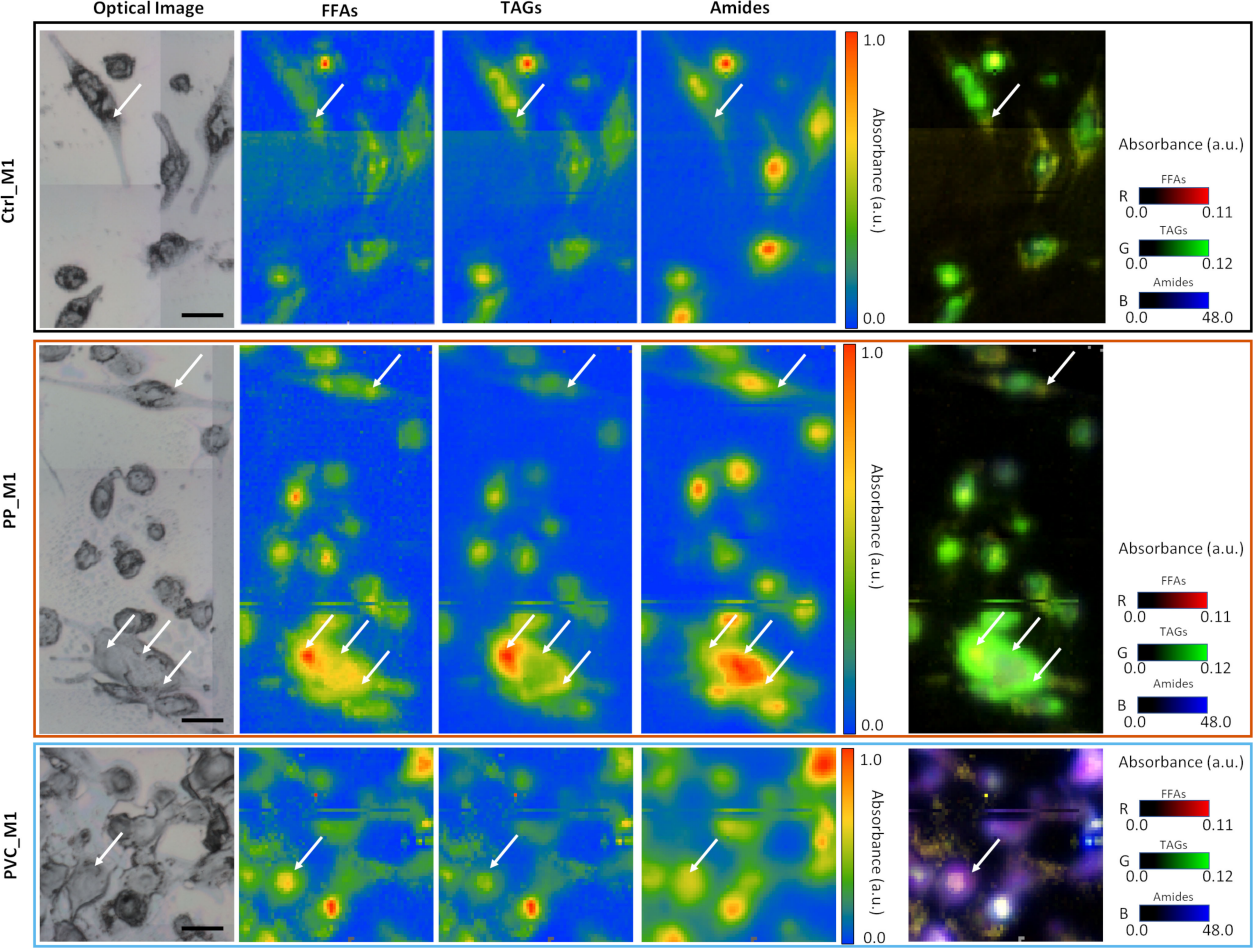
Multipaneled figure representing some of the FTIR images acquired on the three samples. In the first line, the CTRL_M1 cells (black border); in the second line, the PP_M1 cells (orange border); and in the third line, the PVC_M1 cells (blue border). Each row has, from left to right, an optical image of a sub-area of the samples, a chemical image of the free fatty acids (FFAs) obtained integrating the signal at 1,716 cm^−1^, a chemical image of the distribution of triacyclglycerols (TAGs) obtained by calculating the peak height at 1,737 cm^−1^, and the distribution of proteins, obtained by the integration of the amide I and II band in the 1,700–1,480 cm^−1^ range. For all these three (nine) panels, the data have been normalized between 0 and 1 to see differences in distribution of the relative maxima (white arrows). In the fifth panel are placed the RGB composite images of the three channels previously described, not normalized, but presented with a standard scale to better observe the variations of the relative intensities of the three signals. The scale bars are 50 µm.

As previously described in **Figures 6A, B**, PVC_M1 cells are characterized by a lower intensity of the lipidic bands (solid blue line at 3,050–2,800 cm^−1^) concerning the Ctrl_M1 (solid black line), confirmed in **Figure 6D** where the amides-to-lipids ratio is shown. The values of the ratio for PVC_M1 are significantly higher than that of Ctrl_M1. The present evidence can be better explained by observing **Figure 7**: from both optical image and FTIR maps, it is possible to notice that PVC_M1 cells look rounder and slightly thicker in the nuclear area. Instead, the PP_M1 condition reveals a slight yet significant (**Supplementary Table 1**) decrease in the amide/lipid ratio regarding Ctrl_M1. From **Figure 7**, Ctrl_M1 are more adherent and spread onto the SiC windows (our substrate); thus, the decrease of the amide/lipids ratio can be associated with an enhanced lipid synthesis (56). In **Figure 6E**, the boxplot of the carbonyl/lipids ratio demonstrates that PP_M1 are characterized by a slightly higher value of the CO/lipids ratio (check **Supplementary Table 1** for statistical significance of the data) that might be linked to oxidative stress conditions (55, 57, 58). PVC_M1, instead, revealed a lower ratio than that of Ctrl_M1, explained by the lower content of triacylglycerols (TAGs, band height at ~1,737 cm^−1^) for FFAs (band height at ~1,716 cm^−1^). Indeed, in **Figure 6F**, the boxplot of the FFAs/TAGs ratio for the three conditions has the opposite trend of that in **Figure 6E**: the lower value of the mean of PP_M1 (check **Supplementary Table 1** for statistical significance of the data) could be due to a higher formation of TAGs, as confirmed by the Oil Red O staining, whereas PVC_M1 contain more FFAs (check **Supplementary Table 1** for statistical significance of the data). The commented results can be more readily seen in **Figure 7**, where the chemical maps of FFAs and TAGs are displayed for several groups of cells for the three conditions. In Ctrl_M1 and PVC_M1, the two types of fatty acids were comparably distributed and only a few differences can be seen; in contrast, in PP_M1, hotspots of FFAs tend to cluster at the cytoplasmic level, whereas the highest concentration of TAGs tends to accumulate in the cellular outer edge (white arrows in panels in **Figure 7**), as previously observed in the absorption and phase contrast images (**Figure 4D**). These considerations are very evident in **Figure 7**, where the related RGB composite is reported, which combines the three signals mentioned above using the same range of the intensities (see caption) of the three maps of FFAs, TAGs, and amides, normalized from 0 to 1, to favor a direct comparison.

**Figure 8:**
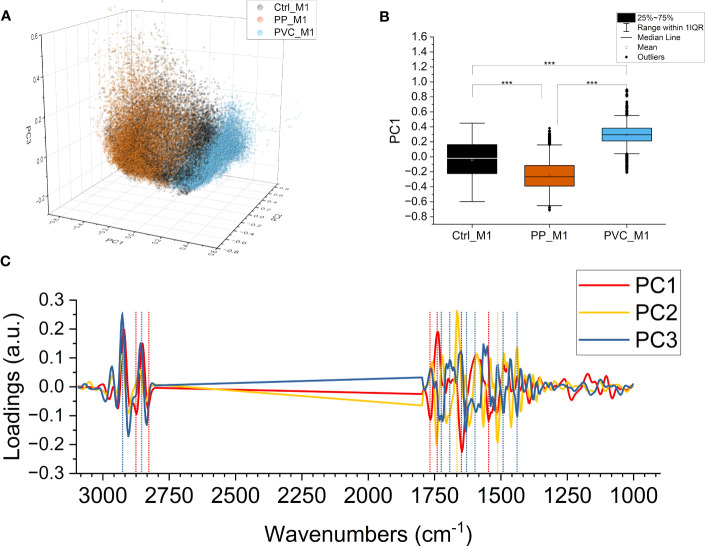
PCA of components PC1, PC2, and PC3. PCA loading of the three components PC1, PC2, and PC3 (47.6%, 12.0%, and 9.3% of the samples’ variance, respectively). Ctrl_M1 are colored black; 20 μg/ml QDs-PP and QDs-PVC exposed macrophage M1 cells are colored orange and light blue, respectively **(A)**. Boxplot of PC1 scores, same color code of the previous panel, ***p < 0.001, black *, and lines indicate significant differences calculated with the Tukey method. Data are presented as box 25%–75% range of the data; whiskers range within the 1st quartile, line: median line, square: mean, black diamonds: outliers **(B)**. PC1 in red, PC2 in yellow, and PC3 in blue; loading vectors were obtained from the PCA. Dashed lines represent each component’s most relevant spectral features **(C)**.

A PCA was performed on the second derivative of the absorbance spectra to evaluate subtler differences induced by the treatments, not only due to variations in the content of specific chemical compounds but also due to conformational or structural modifications. **Figure 8A** presents the 3D scatterplot of the results of the PCA, with the scores plotted along PC1, PC2, and PC3. The Ctrl_M1 are at the center of the graph for all the axes, while PP_M1 leans towards negative values for PC1 and PVC_M1 leans toward positive ones, respectively. The data are separated mainly on PC1, as seen in **Figure 8B**; the graph was obtained by projecting all the points on PC1, allowing for a comparison of the mean values. The outcome revealed that the three distributions are significantly different along PC1, representing the 46.5% of the total variance of the dataset. Thus, the PCA underlines a different response of macrophages according to the composition of NPs. **Figure 8C** illustrates the loading vectors: PC1 in red, PC2 in yellow, and PC3 in blue are presented. The PC1 loading vector contains positive signals from lipids at 2,920 and 2,850 cm^−1^ in the CH stretching region and at 1,737 cm^−1^ of the C=O, meaning that they are higher for PP_M1, average for Ctrl_M1, and lower for PVC_M1. PC1 showed negative protein-related signals at 1,647 cm^−1^ for amide I and at 1,545 cm^−1^ for amide II, meaning that their trend is the opposite of those of the lipids previously described. These protein signals can be assigned to unordered structures (59). Even if of minor amplitude, the three groups also separate along PC2 and PC3, representing 12.7% and 9.3% of the total variance of the dataset, respectively. PC2 mainly separates PP_M1 from the other two groups, and the loading vector has stronger signals in the protein region at ~1,666 cm^−1^, ~1,568 cm^−1^, and the carbonyl signal at ~1,743 cm^−1^.

PC3 instead have the PVC_M1 in the negative hemispace and the PP_M1 in the positive hemispace. The PC3 loading vector contains positive signals from lipids at 2,927 and 2,857 cm^−1^ in the CH stretching region, blue-shifted concerning those of PC1, possibly attributed to less ordered lipids and more fluid membranes (46). The signals in the C=O region are negative instead and are located at ~1,737 cm^−1^ and ~1,716 cm^−1^ of the C=O. PC3 contains protein signals, as well, at 1,632 cm^−1^ for amide I and at 1,490 cm^−1^ for amide II, negative, which can be assigned to β-sheet structures (50, 59).

## 4 Discussion

Although the occurrence of M/NPs has been demonstrated in human blood (60), intestinal tract (61), and immune system (62, 63), the toxicological accumulation mechanism in human cells is still poorly understood. It mainly depends on the difficulties in the identification, localization, and characterization of the polymeric particles due to the analytical interference with macromolecules, such as lipids, proteins, and nucleic acids, especially at small size (nm) and low concentration (μg/ml), making investigation of their related toxicological accumulation rather challenging. For this reason, it is highly demanding for *in vitro* studies to develop both the detection of fingerprints of toxicity and the synthesis/use of model environmental NPs. Most studies are currently using NPs labeled with fluorescent probes (gold NPcs, fluorophores, and quantum dots) (26), which allow the identification of NPs at the cellular level by exploiting the optical properties of the linkers (i.e., fluorescence). The scientific literature rarely reports the synthetic procedures for the most common NPs abundant in the environment (i.e., PP and PVC); indeed, nano- and micro-PS beads are often commercially purchased to conduct most laboratory studies (48, 64). In the present research, we propose using CdSe QDs-labeled PP and PVC NPs as a suitable model for representative environmental NPs, and THP-1 cell line as a surrogate of macrophages (65–67) for immune-toxicological studies.

Cassano et al. already demonstrated that these NPs can be tracked by the bright red fluorescence properties of QDs (32). The light fluorescence microscopy was highly supportive in visualizing the NPs across the cell samples, focusing on the bright, tunable red spots (**Figure 3**, second column). However, a more sensitive and higher-resolution combination of analytical techniques would be highly beneficial to overcome the cellular self-fluorescence, typical of macrophages and exogenous material (68), that interferes with the detection, demonstrating that the use of fluorophores is not always a successful strategy for this aim.

To overcome that, soft X-ray microscopy combined with LEXRF at the TwinMic beamline (Elettra Sincrotrone Trieste, Italy) permits the acquisition of X-ray fluorescence photons of Se, one of the two components labeling NPs, together with the chemical map distribution of low-Z elements (C, O, Na, and Mg) at sub-micron spatial resolution (**Figures 4**, **5**). The simultaneous absorption and phase contrast image acquisition provided additional information on cellular morphology. At such chemical and sub-micron spatial resolution, tracking the NPs and identifying their location across the cells are possible. Observing **Figures 4**, **5**, we can state that NPs of both compositions are uptaken by macrophages and heterogeneously dispersed intracellularly, apparently not forming substantial clusters onto the cell surface, excluding a simple NPs precipitation. Although there is no significant difference in the NPs distribution dependent on the polymer composition, peculiar structural changes were evidenced after 72 h of NP exposure in absorption and phase contrast images.

CdSe-QDs PP NPs seemed to increase bright vesicles in the cytoplasm (**Figure 4C**) or at the plasma membrane level, better realized by the corona formation (**Figure 4D**). In contrast, these bright vesicles were not visible in the CdSe-QDs PVC NPs-treated cells (**Figure 5**), but the presence of depressions across the cytoplasm concerning the control (**Figure 5A**) underlines a different response of macrophages, based on the different polymers that compose the NPs.

Those bright vesicles resulted in LDs by performing a laboratory assay, the Oil Red O staining test (**Figure 2**), indicative of the phagocytosis activation and consequent oxidative stress already demonstrated for this cell model (69). On the other hand, although LDs were not distinctly visible in the PVC-treated cells by phase contrast images, their risen content was inferred by the lipidic test. The accumulation of lipids was quantified by measuring the absorbance at 450 nm after 24–72 h (**Figure 2**). At the 24-h time point, the results interestingly report the gradual increase of lipid contents in accord with the NPs concentration, ranging from 0 to 100 μg/ml, for both NPs. As expected, after 72 h, the enhancement of LDs continued in treated cells. However, above 20 μg/ml concentration of PP and PVC NPs, the number of available macrophages is compromised by the apoptotic process and the higher quantity of NPs at this point starts to interfere with the results. It further confirms the reason beyond utilizing the selected concentration for the present studies. The results suggest the capacity of NPs to enhance the number of LDs, which may be an early stage for further toxicological lipid metabolism impairment, even by not exerting significant cellular cytotoxicity upon NPs incubation (**Figure 1**).

The FTIR technique was exploited to provide unique chemical vibrational information on lipids, protein structure, and nucleic acids at a micrometric spatial resolution (70) to support the abovementioned observations. Based on the spectra in **Figures 6A–C**, it is difficult to directly identify the contributions of the polymeric NPs, which, for example, are expected to have strong signals in the regions of lipids (3,050–2,800 cm^−1^). Therefore, the present work aims to investigate the effect of polymeric materials, by looking for cellular macromolecular changes when in close contact with the NPs. The univariate data analysis showed variations in lipids and protein bands upon incubation with the different NPs (**Figures 6A–C**). By analyzing the chemical images from **Figure 7**, it is possible to correlate how these intensity variations correspond to different distributions of lipid and protein components within the three populations of cells, also reflecting changes in their morphology induced by the treatments. PVC_M1 appear rounder and thicker, whereas Ctrl_M1 and PP_M1 are very adherent; nevertheless, observing the TAGs distribution of this latter group is possible to observe the accumulation of LDs in the outer edge of the cytoplasm. The lipid-related chemical modifications were also evidenced with standard laboratory tests (Oil Red O staining, **Figure 2**) and confirmed by advanced analytical tools such as STXM (**Figures 4**, **5**). The treatment with NPs could cause the cell to respond with increased oxidative processes. From the CO/lipids ratio depicted in **Figure 6E**, a high contribution of CO in PP_M1 cells can be slightly seen; in contrast, a lower ratio in PVC_M1 macrophages is demonstrated, which might be mainly related to augmented FFAs and membrane disruptive processes (71). **Figures 6C, F** better represent the NPs’ impact on the FTIR bands of the carbonyl group at 1,737 cm^−1^. It is a clear evidence of the oxidation of the aliphatic chains of the phospholipidic bilayer at different levels of the oxidation process depending on the different NPs. The chemical formula of the NPs is indeed determinant for the cellular response; the PVC NPs are more hydrophobic than the PP NPs, due to the presence of the halogen (-Cl), which protects the NPs from a potential hydroxyl radical attack (72, 73); thus, it seems to attack the cellular membranes preferably. Based on that, the PVC_M1 curve in **Figure 6C** demonstrates the consequent effect in the increase of aldehyde and ketone products (1,716 cm^−1^).

The PP_M1 curve suggests more advanced oxidative stress that leads to the formation of CO in the cellular structures, a process confirmed by the high absorption band typical of a carboxyl group (1,737 cm^−1^) (47).

From the multivariate analysis, all the variations in the second-derivative spectra are considered at one glance. Therefore, it is possible to observe the finer changes in the macromolecule peaks that the NPs cause. From the scatterplot of the PCA in **Figures 8A, B**, it is possible to conclude that the two types of NPs certainly affect the cells but cause different responses, as can be seen by the clear separation of the three datasets along PC1, with the controls in the center, visible in **Video 1**. This component represents, at the same time, variations in lipid content and structure and protein folding since it can be seen in the increase of random structures and β-sheet aggregates in treated cells, signals that often correlate with cellular suffering (74) and apoptotic processes (71).

In conclusion, the advanced analytical techniques, XRF, accurately detected the NPs internalization across the macrophages at high spatial resolution and evidenced morphological changes, further analyzed by staining-based laboratory techniques. The chemical changes related to lipid metabolism impairment induced by NPs were better resolved by FTIR spectromicroscopy.

Based on the present results, a deeper study will be conducted to characterize the NPs–lipid and NPs–protein interactions approaching lipidomic and proteomic technologies.

Moreover, the advanced multi-technique approach presented here will be a model for future *in vitro* investigations focused on more specific accumulation effects of prenatal and postnatal M/NPs exposure.

## Data availability statement

The datasets reported in **Figures 4**, **5** for this study can be found in the Elettra Sincrotrone Trieste repository: https://doi.datacite.org/dois/10.34965%2Fi10862. The other data are available upon request.

## Author contributions

FZ, AG, LP, and GC conceived the study. DC and RLS synthesized and characterized the NPs. FZ prepared the cell samples with CA support and generated the related graphs. FZ, AG, and VB performed the analyses at TwinMic Beamline, and FZ and GB performed those at SISSI Beamline at Elettra Sincrotrone Trieste and generated the related plots. FZ wrote the original draft, supervised by AG, LP, and GB. GR contributed to the draft. All the authors have read and agreed to the published version of the manuscript. All authors contributed to the article and approved the submitted version.

## Supplementary material

Supplementary Video 1 3D representation of the PCA scatterplot rotating around the vertical axis. Video created with Origin Pro 2023.
