# Supplementary material for: Morphological and lipid metabolism alterations in macrophages exposed to model environmental nanoplastics traced by high-resolution synchrotron techniques
Source: Front Immunol. 2023 Sep 8;14:1247747. doi: 10.3389/fimmu.2023.1247747 (PMC10515218; doi:10.3389/fimmu.2023.1247747)
Supplement: Supplementary file 1 [file Table_1.docx]

# Supplementary Tables

**Supplementary table 1.** Tables reporting the results of the ANOVA analyses for the selected bands’ ratio amide/lipids **A)**, CO/lipids **B)**, FFAs/TAGs **C)**, used to generate the boxplots in figure 6 **D-F**. (SD: standard deviation, SE: standard error, ***: p value ≤ 0.001)


| **Sample name** | **Mean** | **SD** | **SE of Mean** | **Significance** |
| --- | --- | --- | --- | --- |
| Ctrl_M1 | 1.9324 | 1.07902 | 2.074⋅10^-2^ | *** |
| PP_ M1 | 1.45807 | 0.75558 | 7.42⋅10^-3^ | *** |
| PVC_ M1 | 3.52528 | 1.17231 | 1.175⋅10^-2^ | *** |


| **Sample name** | **Mean** | **SD** | **SE of Mean** | **Significance** |
| --- | --- | --- | --- | --- |
| Ctrl_ M1 | 0.13871 | 0.05845 | 1.12⋅10^-3^ | *** |
| PP_ M1 | 0.15206 | 0.04302 | 4.22696⋅10^-4^ | *** |
| PVC_ M1 | 0.03447 | 0.02141 | 2.14638⋅10^-4^ | *** |


| **Sample name** | **Mean** | **SD** | **SE of Mean** | **Significance** |
| --- | --- | --- | --- | --- |
| Ctrl_ M1 | 0.62756 | 0.19848 | 3.82⋅10^-3^ | *** |
| PP_M1 | 0.54593 | 0.25694 | 1.54⋅10^-3^ | *** |
| PVC_ M1 | 1.181 | 0.20791 | 2.08⋅10^-3^ | *** |
